# Is Autonomic Modulation Different between European and Chinese Astronauts?

**DOI:** 10.1371/journal.pone.0120920

**Published:** 2015-03-23

**Authors:** Jiexin Liu, Yongzhi Li, Bart Verheyden, Shanguang Chen, Zhanghuang Chen, Yuqing Gai, Jianzhong Liu, Jianyi Gao, Qiong Xie, Ming Yuan, Qin Li, Li Li, André E. Aubert

**Affiliations:** 1 Department of Cardiology, Beijing Friendship Hospital, China Capital Medical University, Beijing, China; 2 Department of Cardiology, University Hospital Gasthuisberg, K. U. Leuven, Leuven, Belgium; 3 China Astronaut Center, Beijing, China; University of Hull, UNITED KINGDOM

## Abstract

**Purpose:**

The objective was to investigate autonomic control in groups of European and Chinese astronauts and to identify similarities and differences.

**Methods:**

Beat-to-beat heart rate and finger blood pressure, brachial blood pressure, and respiratory frequency were measured from 10 astronauts (five European taking part in three different space missions and five Chinese astronauts taking part in two different space missions). Data recording was performed in the supine and standing positions at least 10 days before launch, and 1, 3, and 10 days after return. Cross-correlation analysis of heart rate and systolic pressure was used to assess cardiac baroreflex modulation. A fixed breathing protocol was performed to measure respiratory sinus arrhythmia and low-frequency power of systolic blood pressure variability.

**Results:**

Although baseline cardiovascular parameters before spaceflight were similar in all astronauts in the supine position, a significant increase in sympathetic activity and a decrease in vagal modulation occurred in the European astronauts when standing; spaceflight resulted in a remarkable vagal decrease in European astronauts only. Similar baseline supine and standing values for heart rate, mean arterial pressure, and respiratory frequency were shown in both groups. Standing autonomic control was based on a balance of higher vagal and sympathetic modulation in European astronauts.

**Conclusion:**

Post-spaceflight orthostatic tachycardia was observed in all European astronauts, whereas post-spaceflight orthostatic tachycardia was significantly reduced in Chinese astronauts. The basis for orthostatic intolerance is not apparent; however, many possibilities can be considered and need to be further investigated, such as genetic diversities between races, astronaut selection, training, and nutrition, etc.

## Introduction

Since the start of human spaceflight, more than 50 years ago, it was found that microgravity is associated with many adaptations in the cardiovascular system [1–4]. Space flight is characterized by an absence of gravitational stress, which results in various imperative cardiovascular modifications, such as body fluid redistribution [5,6], cardiac atrophy [7,8], vascular remodeling [9,10], and dynamic cardiovascular regulation [11–13]. After return to Earth, microgravity-induced adaptations cause a range of physiologic problems for astronauts because of return to normal gravitational stress. One of the most important manifestations is the variable degree of post-spaceflight orthostatic intolerance [14] (i.e., the inability to stand for prolonged periods with a risk of fainting and the occurence of tachycardia).

From an operational point of view, post-spaceflight orthostatic intolerance can be considered as one of the most problematic conditions, i.e. autonomic egress of the space ship after landing can be jeopardized. Previous studies have shown that although blood pressure (BP) can be well-maintained, post-flight tachycardia occurs in all European astronauts on the first several days after landing, especially in the standing position [1]. Although many studies have focused on this impaired orthostatic tolerance, the underlying mechanisms are unclear. In contrast, we found recently that heart rate (HR) is also well-maintained in Chinese astronauts after short-duration space travel, even in the standing position. Thus, it can be inferred that there are some differences in the autonomic cardiovascular control mechanisms between European and Chinese astronauts. It is therefore necessary to compare the possible differences in autonomic modulation between European and Chinese astronauts before and after short-duration spaceflight.

In the current study we tested the hypothesis that post-spaceflight tachycardia is related to the degree of adaptations in cardiovascular autonomic control, with higher sympathetic dominance and lower vagal-cardiac modulation. The purpose of the current study was to investigate the differences in cardiovascular and respiratory control, as well as the arterial baroreflex function between two groups of European and Chinese astronauts, while attempting to identify the determinants underlying post-spaceflight orthostatic intolerance.

## Methods

### Subjects

This study was conducted during three scientific ESA-Soyuz missions (Odissea, Cervantes, and Delta: 10–11 day missions provided by the European Space Agency [ESA]) and two Chinese Shenzhou missions (Shenzhou 6 & 7: 3–5 day spaceflights), provided by the Chinese Space Agency. Five male European astronauts (age, 40–52 years; height, 169–185 cm; weight, 67–90 kg), and five male Chinese astronauts (age, 40–42 years; height, 168–172 cm; weight, 62–70 kg) were studied before and after spaceflight. The baseline characteristics of the astronauts are shown in Table 1.

**Table 1:** General characteristics of the European and Chinese astronauts.

	Pre-flight				Post-flight		
					*R+1*	*R+4*	*R+10*
**European astronaut**	**Age (years)**	**Length (mm)**	**Body weight (kg)**	**BMI (kg/m** ^**2**^)	**BMI (kg/m** ^**2**^)	**BMI (kg/m** ^**2**^)	**BMI (kg/m** ^**2**^)
1	45	185	90	26.3	25.4	26.0	26.3
2	41	181	64	19.5	17.7	18.6	19.5
3	40	180	68	21.0	20.1	20.4	21.0
4	40	182	81	24.5	23.8	24.2	24.5
5	40	175	81	27.1	26.1	26.7	27.1
*mean (SD)*	*41 (2)*	*180 (4)*	*77 (11)*	*23*.*7 (3*.*3)*	*22*.*6 (3*.*6)* ^#^	*23*.*2 (3*.*5)* ^#^	*23*.*7 (3*.*3)*
**Chinese astronaut**	**Age (years)**	**Length (mm)**	**Body weight (kg)**	**BMI (kg/m** ^**2**^)	**BMI (kg/m** ^**2**^)	**BMI (kg/m** ^**2**^)	**BMI (kg/m** ^**2**^)
1	40	170	64	22.1	21.5	21.8	22.1
2	41	172	70	23.7	23.0	23.3	23.7
3	42	172	66	22.3	21.6	22.0	22.0
4	42	168	62	22.0	21.3	22.0	22.0
5	42	172	64	21.6	20.3	21.0	21.3
*mean (SD)*	*41 (1)*	*171 (2)* *	*65 (3)* *	*22*.*3 (0*.*8)*	*21*.*5 (1*.*0)* ^#^	*22*.*0 (0*.*8)* ^#^	*22*.*2 (0*.*9)*

BMI (body mass index) = weight (kg) / height^2^ (m^2^); R+1 = the first day after landing, R+4 = the 4^th^ day after landing, R+10 = the 10^th^ day after landing;

* p < 0.05 compared to European astronauts.

^#^ p< 0.05 compared to pre-flight baseline

All subjects were in excellent health, without any history of chronic or recent acute illnesses. During these short duration flights, no routine physical exercise program was performed as a counter-measure. Each subject was thoroughly briefed on the experimental procedures prior to giving written consent. Medications, cigarette smoking, and alcohol- and caffeine-containing drinks were not permitted during the study in the pre-and post-flight stages.

### Study protocol

The study protocol has been extensively described in the papers by Verheyden et al [6,15,16]. In the following paragraphs a short version is described. Pre-flight data was collected from all astronauts 21–30 days before launch (pre-flight); post-flight data was collected 1, 4, and 10 days after return to Earth (R+1, R+4, and R+10, respectively). All tests on the European astronauts were carried out in the Medical Building of the Gagarin Cosmonaut Training Center (Moscow, Russia), and all tests on the Chinese astronauts were carried out in the China Astronaut Training and Research Center (CATRC; Beijing, PR of China).

Dedicated software was used allowing standardization of each test procedure [17]. All recordings were measured in a temperature-controlled room (24°C) in the morning before 12:00 h, starting with the subjects resting quietly in the supine position with comfortable spontaneous respiration for 10 min (baseline). Then, the subjects were instructed to pace their breathing to an audio/visual stimulus. Paced breathing was maintained for 3 min, and was performed in succession in which respiratory sequences were evenly spaced in time at a preset rate of 12 breaths min^−1^ or 0.2 Hz. This was followed by a standing pattern, which included 10 min of spontaneous breathing while standing (baseline) and 3 min of paced breathing recording of 12 breaths min^−1^ or 0.2 Hz in the standing position.

The experimental protocol was approved by the Medical Ethical Committee of the University Hospital Gasthuisberg (K.U. Leuven, Belgium) and the Medical Ethical Committees of the ESA and CATRC.

### Data acquisition and analysis

The analytic methods have been described in detail in previous works [15,16,18,19]. In brief:

The following parameters were recorded:

- the ECG (Medtronic 9690; Minneapolis, MN, USA)
- beat-to-beat finger arterial BP (Portapres Model-2; FMS, Amsterdam, The Netherlands
- Brachial arterial BP (STBP-780’ Colin, Komaki, Japan) was measured three times in each positione

After peak detection on the ECG, an R-R interval (RRI) time series was obtained.

The HR was computed from the ECG recording. Systolic arterial (SAP) and diastolic arterial pressures (DAP) were derived from the arterial pressure waveform. The mean arterial pressure (MAP) was calculated as the true integral of the pressure wave over one beat, divided by the corresponding beat interval.

During the 10-min baseline recording, time-domain analysis of spontaneous baroreflex sensitivity (BRS) was performed using the cross-correlation method [20]. Two complementary aspects of arterial baroreflex function were assessed: 1) BRS, which provides qualitative information; and 2) number of BRS estimates, which provides quantitative information.

During the 3-min paced breathing protocol, beat-to-beat SAP and the RRI time series were constructed for frequency analysis[21]. using Fast Fourier transform. Respiratory powers were expressed as the area under the spectrum from 0.18 to 0.22 Hz and used as a marker of respiratory sinus arrhythmia (RSA). A second spontaneous rhythm occurring over an approximate 10-s cycle and the resulting low-frequency band (0.04–0.15 Hz) was obtained as well for SAP variability [16]. Power spectral units for RRI and SAP fluctuations were squared amplitudes.

### Statistical analysis

Software for statistical analysis was SPSS (version 13.0 for Windows; Scientific Packages for Social Sciences, Inc., Chicago, IL, USA). Data are given as the mean ± SD. The bias between brachial and finger MAP readings was estimated by calculating the mean difference and the standard deviation of individual differences over different recording sessions [22]. Normal distributions were approximated for spectral data by logarithmic transformations. Postural differences in all parameters before and after spaceflight were evaluated by paired t-tests. Parameters were analyzed across sessions using multivariate repeated-measures ANOVA; the non-parametric Wilcoxon text was used to evaluate changes at each time point as compared to baseline. The differences between the two groups in the same position were compared using independent samples t-tests.

Pre- and post-flight standing and supine data were used to assess functional operational curves by means of linear regression analysis with 95% confidence intervals. Graphical analysis was followed by hypothesis testing (univariate analysis of variance) to compare pre-flight data to post-flight standing and supine reference values with multiple contrast analysis at a 0.05 significance level. P-values <0.05 were considered statistically significant.

## Results

Table 1 shows the general characteristics of all astronauts. No significant difference existed in the ages between European and Chinese astronauts. The mean height and weight were less in Chinese astronauts compared to European astronauts. No significant difference existed in the body mass index (BMI) between the two groups before and after spaceflight; although the BMI decreased in the two groups after spaceflight, the BMI returned to the pre-flight baseline level after a 10-day recovery.

**Fig 1:**
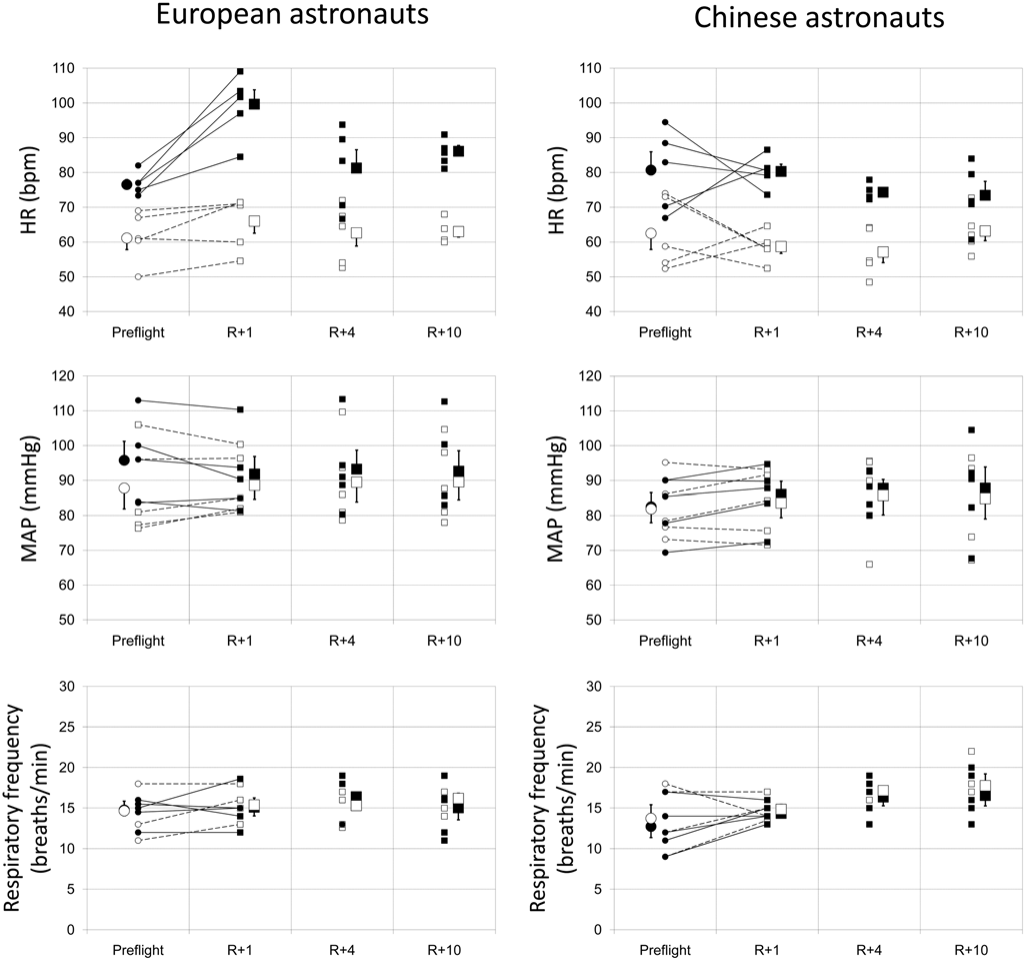
Evolutions in heart rate (HR; top), mean arterial pressure (MAP; middle), and respiratory rate (bottom) before and after spaceflight in the European and Chinese astronauts in the supine and standing position. R+1 = the first day after landing; R+4 = the 4^th^ day after landing; R+10 = the 10^th^ day after landing. ○ (small open circle) baseline individual data in supine position; ● (small black filled circle) baseline individual data in standing position; □ (small open spuare) post-flight individual data in supine position; ■ (small black filled square) post-flight individual data in standing position; ○ (large open circle) baseline mean value in supine position; ● (large black filled circle) baseline mean value in standing position; □ (large open square) post-flight mean value in supine position; ■ (large black filled square) post-flight mean value in standing position.

Fig. 1 presents the detailed changes in the HR, MAP, and respiratory rate in European (left panel) and Chinese astronauts (right panel) from pre-flight to 10 days post-flight. The individual and mean values from pre-flight baseline to 10 days post-flight are shown in the supine and standing positions, with the individual transitions from pre-flight baseline to the first measurement post-flight (R+1 [the first day after landing]). No significant difference existed in any of these parameters between European and Chinese astronauts pre-flight. In the supine position, the HR, MAP, and respiratory rate were well maintained post-flight; no significant difference existed compared to pre-flight values in the two groups. In the standing position, the orthostatic MAP and respiratory rate were well-maintained in the two groups. All five European astronauts had a large increase in HR at R+1; specifically, the HR was >100 beats per minute (bpm) in three astronauts, with the highest HR = 109 bpm. The mean post-flight HR was significantly higher compared to pre-flight (p = 0.041). The post-flight HR in Chinese astronauts was well-maintained in the standing position. Only two of five Chinese astronauts had an increase in HR, while the highest HR at R+1 was 87 bpm. No significant difference existed in the mean HR pre- and post-flight in the Chinese astronauts.

**Fig 2:**
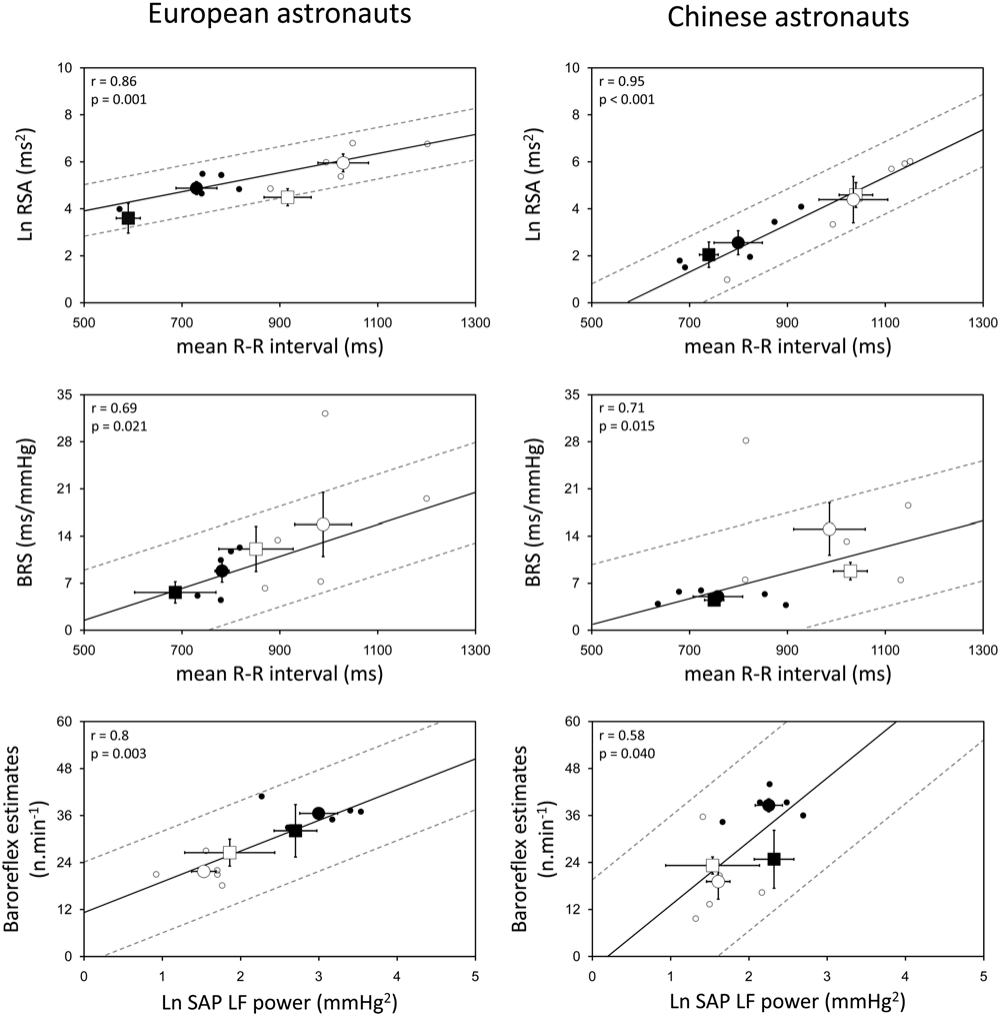
Physiologic relationships pre- and post-flight. Relationships between respiratory sinus arrhythmia (RSA) & R-R interval (upper), baroreflex sensitivity (BRS) & R-R interval (middle), baroreflex estimates & LF power of systolic arterial pressure (SAP; lower) before and immediately after spaceflight in the European and Chinese astronauts in supine and standing positions. Spectral data (logarithmically-transformed) were analyzed during paced breathing at 0.2 Hz. Full lines show the linear correlation of all individual data (in supine and standing positions) during pre-flight baseline; broken lines show the 95% confidence intervals. Post-flight data points (squares) are scattered along the regression line to evaluate the adaptation to return of gravity. ○ (small open circle) baseline individual data in supine position; ● (small black filled circle) baseline individual data in standing position; ○ (large open circle) baseline mean value in supine position; ● (large black filled circle) baseline mean value in standing position; □ (large open square) post-flight mean value in supine position; ■ (large black filled square) post-flight mean value in standing position.

Fig. 2 shows the respiratory modulation of RRI (RSA) and cardiac BRS as a function of the mean RRI, and the number of cardiac baroreflex estimates as a function of the baroreceptor input, i.e., the low frequency (LF) component of SAP variability (~10-s cycle intervals), collected in the standing and supine positions before and after spaceflight (R+1) data recording sessions in European (left panel) and Chinese astronauts (right panel). The RSA was derived from RRI times series obtained during the 3-min paced breathing (0.2 Hz), while the BRS was calculated during spontaneous respiration over the 10-min baseline recordings. Pre-flight individual scores are scattered together with the mean values, showing a functional reduction in the mean RRI upon standing that was significantly related to a decrease in RSA and a lower cardiac BRS, as well as a rise in the LF power of SAP upon standing, which resulted in more baroreflex estimates compared to the supine position in European and Chinese astronauts. In the supine position, the RSA and LF power of SAP were similar in European and Chinese astronauts, while in the standing position, values were higher in European compared to Chinese astronauts (RSA, 152 ± 82 ms^2^ vs. 22 ± 24 ms^2^, p = 0.016; LF power of SAP, 22 ± 11 vs. 10 ± 4 mmHg^2^, p = 0.034). The BRS and baroreflex estimates were similar in the two groups in the supine and standing positions. On R+1 compared to the pre-flight values, the RSA had decreased significantly in the supine position (p = 0.022) and showed a tendency to decrease in the standing position (p = 0.112) in European astronauts, and remained at that lower level until 10 days after landing (R+10). No significant change was observed in the RSA in Chinese astronauts after spaceflight. In no subjects, the BRS, baroreflex estimates, and LF power of SAP showed a significant change after astronauts returned to Earth.

## Discussion

Although it has been reported that removal of gravitational stress during spaceflight alters physiologic adaptive mechanisms and reduces the orthostatic tolerance in astronauts after spaceflight [2,14–16], most results are based on the data from the National Aeronautics and Space Administration (NASA) in the US, the ESA astronauts, and Russian cosmonauts. This paper adds significant data about manned Chinese spaceflights.

This is the first study to compare differences in cardiovascular autonomic regulation and respiratory control before and after short-duration space missions in astronauts of different races, with different training methods, different cultural backgrounds, and different nutritional habits. A major strength of this study was the explicit effort in obtaining uniform data across different spaceflight missions of the two agencies (ESA and CATRC) by applying standardized experimental procedures using a computer-guided protocol [17]. The main findings of our study were as follows: (1) pre-flight cardiovascular autonomic nervous control in European astronauts upon standing was based on a higher vagal and higher sympathetic balance compared to Chinese astronauts; and (2) post-flight orthostatic tachycardia was more pronounced in European astronauts, possibly induced by a depression in vagal cardiac control, which was not observed in Chinese astronauts.

### Baseline autonomic characteristics

As introduced in our previous work, the dynamic relationships between qualitative components of vagal-cardiac modulation and the mean RRI as a reference were used to assess the re-adaptation to gravity after return to Earth (Fig. 2). This type of analysis is warranted because the mean RRI has been identified as a major determinant of overall HR variability [15,23]. Similarly, the effectiveness of cardiac baroreflex modulation in driving the sinoatrial node during recovery was investigated as a function of the dynamic input–output relationship (Fig. 2, lower part) [15].

The present data indicate that autonomic modulation appears different between European and Chinese astronauts before exposure to microgravity. Pre-flight standing vagal modulation and sympathetic nervous activity were significantly higher in European astronauts than Chinese astronauts, despite a comparable baseline HR and arterial pressure. This phenomenon is difficult to explain with the present limited data derived from a small number of subjects; however, the possible explanations include the following: 1. different personal characteristics with different genetic diversities between different races [24–27]; 2. differences in body habitus between Chinese and Europeans populations [15,28–31]; 3. selection of astronauts (the Chinese were selected from fighter pilots only, while the Europeans were selected from a mix of scientists and pilots); 4. training methods [32–34] (none of the astronauts on these short-duration missions submitted to counter-measures during spaceflight); and 5. nutrition [35].

The only difference we identified between European and Chinese astronauts in baseline characteristics was the significant difference in height; however, little data are available that suggest standing results in higher vagal modulation and nervous sympathetic activity in taller people, possibly related to larger intravascular hydrostatic pressure gradients [36]).

### Post-flight orthostatic tachycardia

Standing upright immediately after spaceflight is difficult for most astronauts. In many previous studies from NASA, ESA, and Russia, post-flight orthostatic tachycardia has been commonly reported, while HR has remained stable in the supine position compared to pre-flight [1,16,37]. This finding was thought to be due to a post-flight sympathetic dominance of HR control indicated by a significant decrease in high-frequency power of heart rate variability (HRV) compared to pre-flight values [1] and increased levels of noradrenaline [38–41], as well as increased human muscle sympathetic nerve activity [42–44] after spaceflight.

In the current study we noted orthostatic tachycardia in all five European astronauts on R+1, but only two of five Chinese astronauts showed an increase in HR after the space mission (Fig. 1, upper part). Although the flight durations were slightly different between the European and Chinese astronauts, flight duration is unlikely to be a determinant for post-flight orthostatic tachycardia [45] because the effects of microgravity are felt immediately [1,6]. In agreement with the results of Verheyden et al. [16], we observed a reduction in RSA after spaceflight in the European astronauts in the supine and standing positions, which indicated a post-flight vagal cardiac neural deficit; however, no vagal cardiac depression was observed in the Chinese astronauts in the supine or standing position. This result is also difficult to explain and needs to be investigated further.

Cardiac baroreflex function after spaceflight remains to be elucidated. In several previous studies, it was reported that the hypotensive buffering of baroreflex response was affected [13,46], while the hypertensive buffering remained intact [40]. Nevertheless, the spontaneous baroreflex, including both hypotensive and hypertensive buffering, as measured by Sigaudo-Roussel et al. [47], did not show significant differences after spaceflight during standing. In agreement with Sigaudo-Roussel et al. [47], we found that the baroreflex sensitivity was well-maintained after spaceflight in all subjects.

Similar to the post-spaceflight orthostatic tachycardia, patients with postural orthostatic tachycardia syndrome (POTS) have problems maintaining consciousness when changing position. In a study published in 2010, Fu et al. [48] found that despite intact autonomic function in POTS patients, marked tachycardia during orthostasis was attributable to a small heart coupled with reduced blood volume, while exercise training improved or even cured this syndrome in most patients. This finding implies that cardiac size and volume might be important determinants underlying post-spaceflight orthostatic tachycardia; however, weight loss was similar in both groups after landing.

It has been reported that respiratory frequency decreases in space [49–51], which might be related to the reduction in lung volume [50], tidal volume, pulmonary ventilation, and metabolic rate [49]. In a long-duration space mission study, Baevsky et al. [51] observed that the respiratory rate decreased to values close to 6 breaths per minute in some cosmonauts after 5 months in space; the respiratory rate rapidly recovered or even exceeded the baseline values after return to Earth. In the current study, we did not find any significant changes in respiratory rate after spaceflight in European [52] or Chinese astronauts. This finding implies that physiologic deconditioning due to microgravity in pulmonary function may recover rapidly, which might not be the basis for post-spaceflight orthostatic tachycardia.

There is also substantial evidence from the literature that initial adaptations of fluid regulation and neural cardiovascular mechanisms in space occur more rapidly than over a 5-day period. The following cardiovascular events have been reported to take place immediately after entering weightlessness. First, the acute mechanical consequences of microgravity lead to prompt cardiac distention [53]. Increased cardiac filling early in-flight is confirmed by an elevated stroke volume and cardiac output [49,54]. The higher stroke volume and cardiac output triggers a fast depressor reflex that decreases the HR and BP at once [54]. Second, right atrial stretching enhances the secretion of atrial natriuretic peptide [55], leading to increased vascular permeability, which together with increased transmural pressure, facilitates extravasation of fluid and sodium [56]. In addition, the expanded central blood volume is sensed as a ‘fluid-volume overload’ and might inhibit the renin–angiotensin system [57]. Third, it should be noted that some plasma volume loss may already precede launch because astronauts commonly experience ≥2 h in a supine legs-elevated posture prior to launch [58]. Also, space motion sickness and diminished fluid intake in the first days of microgravity exposure may contribute to an early plasma volume loss [59].

All of the above cardiovascular effects of microgravity take place rapidly within 1–3 days in space [60]. Di Rienzo et al. [61] showed that HR baroreflex sensitivity and markers of cardiac vagal modulation tend to be increased during the first hours of spaceflight and return to baseline in subsequent flight phases. Most likely, the dynamic adaption of integrative cardiovascular neural control takes place more rapidly, following the pattern of fluid volume regulation, i.e., within 1–3 days in space.

## Limitations

One of the most prevalent limitations of life science experiments carried out in space concerns the small sample size (4–6 subjects) in most prior investigations. In our study we included 10 subjects taking part in five different space missions with spaceflights of similar duration, and used the exact same experimental methods. The small number of subjects is a general problem of space-related research. Nevertheless, the data show a clear influence on cardiovascular autonomic control mechanisms between European and Chinese groups induced by spaceflight. Another limitation inherent to spaceflight examinations is the wide range of parallel experiments that might yield confounding influences on the study outcome. In an attempt to control for most of these side effects, we have imposed strict standardization of experimental conditions using a computer-guided protocol across different missions. Fluid intake and nutrition before and after the flight could not be controlled.

## Conclusion

Before spaceflight, no differences in the baseline cardiovascular and respiratory characteristics were observed between the European and Chinese astronauts. Pre-flight baseline standing sympathovagal balance in the European astronauts was based on higher values of both vagal and sympathetic modulation. Post-flight orthostatic tachycardia in the European astronauts appeared to be mainly due to a reduction in cardiac vagal modulation, which was not observed in Chinese astronauts.
